# Geometry assessment of ultra-short pulsed laser drilled micro-holes

**DOI:** 10.1007/s00170-020-06199-5

**Published:** 2020-10-23

**Authors:** Matthias Putzer, Norbert Ackerl, Konrad Wegener

**Affiliations:** 1 inspire AG, Technoparkstrasse 1, Zurich, Switzerland; 2 Department of Mechanical Engineering, IWF, ETH Zurich, Leonhardstrasse 21, Zurich, Switzerland

**Keywords:** Ultra-short pulsed laser, Micro-holes, Micro-computed tomography, Circular path drilling

## Abstract

Ultra-short pulsed laser ablation enables a defined generation of micro-holes. A parameter study on the ablation characteristics of copper clearly reveals a benefit for green wavelength with lower threshold fluence, simultaneously increasing the Rayleigh length. The use of a circular drilling method allows a defined manufacturing of micro boreholes and micro through-holes with 35 μm diameter of up to 165 μm and 300 μm length. Introducing high-resolution micro-computed X-ray tomography studying the micro-hole evolution and adjacent geometrical transformations reveals micrometer resolution and high usability. The conical geometry evolving up to an aspect ratio of 5:1 fits well to established models known for percussion drilling. However, increasing the number of pulses leads to non-conical geometry evolution, and this resulting geometry is studied for the first time. Henceforth, the exact geometrical evolution from conical to cylindrical shape upon laser drilling can be resolved revealing the impact of multiple reflections at the generated steep flanks.

## Introduction

In recent years, ultra-short pulsed laser (USP) machining is becoming a viable technology for industrial application [1]. The equipment costs have been constantly decreasing, and several benefits are leading to a strong increase in usage and application in industry.

Laser pulses from such USP systems have a typical pulse duration of picoseconds to femtoseconds reaching power densities higher 10^12^ W cm^−2^, which causes a non-linear and/or multi-photon absorption in the radiated materials. A thin and small volume on the surface gets ablated by the absorbed energy, and due to the quick heating and cooling, the heat-affected zone (HAZ) is narrow whereby damage in the underlying material is negligible [2]. The small lateral size of the focused spot being several tens of square micrometers allows texturing the surface in the micrometer range, and the interaction of linear-polarized light with the matter leads to hierarchical structures in the nanometer size known as laser-induced periodic surface structures (LIPSS).

Due to the low damage of the substrate below the absorbing and ablated material, structural modification and ablation of hard-to-machine and heat-sensitive materials is feasible. These include ceramics like alumina, zirconia [3–5], or glass [6, 7]. In addition, the machining of diamond and cubic boron nitride without phase transition is possible [8, 9]. The negligible HAZ and forceless manufacturing makes the production of work pieces or geometries in the micron size possible [10]. Moreover, using an iterative approach with an optical measurement step enables higher precision accompanied by lower manufacturing time [11].

Laser drilling is a method to drill especially holes in the micro-range with highly defined shape, zero-taper geometry [12], and high-aspect ratio [13, 14]. Compared with mechanical drilling, laser drilling offers a broader variety of possible strategies. The simplest one is single shot drilling at high fluence, which is often used for non-critical shape requirements, where several thousand holes per second are practicable [15]. For high aspect-ratio holes, one laser shot does not ablate deep enough, so the use of multiple pulses at the same location is applied, which is termed percussion drilling [16]. Another approach is a circular motion of the laser beam during drilling that leads to highly accurate hole geometries with high-aspect ratio, where the rotation speed of the beam around a fixed axis can be up to 30,000 rpm. Different ways are applicable; one is keeping the focus at the initial height, while the laser spot follows a circular trajectory during drilling, which is called trepanning. Additionally, changing the focus in a continuous or stepwise manner directs the laser spot following a downward helical trajectory, called helical drilling. With these processes, it is only possible to drill micro-holes, which are conical, whereby the taper angle is positive. The most advanced process technique offers drilling cylindrical holes or even holes with a positive taper angle [12]. The beam is controlled using special optics allowing to tilt the beam with an inclination of up to ± 5° forming holes with no or negative taper angle [17].

Micro-holes play a significant role in the function of various components in machines and other systems. Laser drilling is suitable for this purpose in various industrial manufacturing processes. Compared with conventional methods using physical drills or wires, there is no wear of the tools. In addition, the same tools can be used to machine a variety of materials, including difficult to machine and heat-sensitive workpiece materials.

In the field of machining, it would be advantageous to guide the cooling lubricants in a defined manner via wetting gradients over holes, which are produced by laser drilling [18]. The channels in injection nozzles as well as in turbine blades are critical and function determining for the operation of the component in the corresponding environment. An optimal flow of fuel or cooling air is the subject of many developments, whereby laser drilling offers new approaches. This allows the bore geometry to be improved, production optimized, and damage to materials reduced [19, 20]. The bleaching or removal of near-surface black inclusions in diamonds by injecting strong acids through tiny laser-drilled channels is a common method to increase the quality and clarity of diamonds [21]. Micro-holes are a functional feature in microelectronics serving as microelectrodes or via connections through printed circuit boards, where a high-aspect ratio is important and a stack of dissimilar layers of material present [22]. In order to produce solar cells with higher efficiencies, a new design and process chain have been developed utilizing laser drilled through-holes, which are metallically contacted to the backside [23, 24]. In the pharmaceutical and biotechnological industry, holes are also crucial for the performance of various functions. Here, the generation of holes by laser drilling is a widely used method. An example is a polymer membrane with through-holes leading to a special wettability showing improved oil-water separation [25]. Furthermore, a membrane with specially designed holes has shown the possibility of unidirectional gas transport [26]. Laser-drilled micro-holes in pills, which due to their special structure serve as an osmotic drug delivery system, guarantee a time- and quantity-controlled release of the medication [27]. Recently, it has been confirmed that micro-holes and micro-bores, drilled by an USP laser, can function as micro-reactors for CO_2_ reduction reactions in electrocatalysis. Due to their size and geometry, a proton gradient is formed after connection to an electrical source, in which carbon dioxide is converted into hydrocarbons [28].

## Experimental details

### Material and machine

In this study, oxygen-free copper (OFC) platelets of high purity (99.95% Cu) are utilized in the as-received surface condition after rolling.

**Fig. 1:**
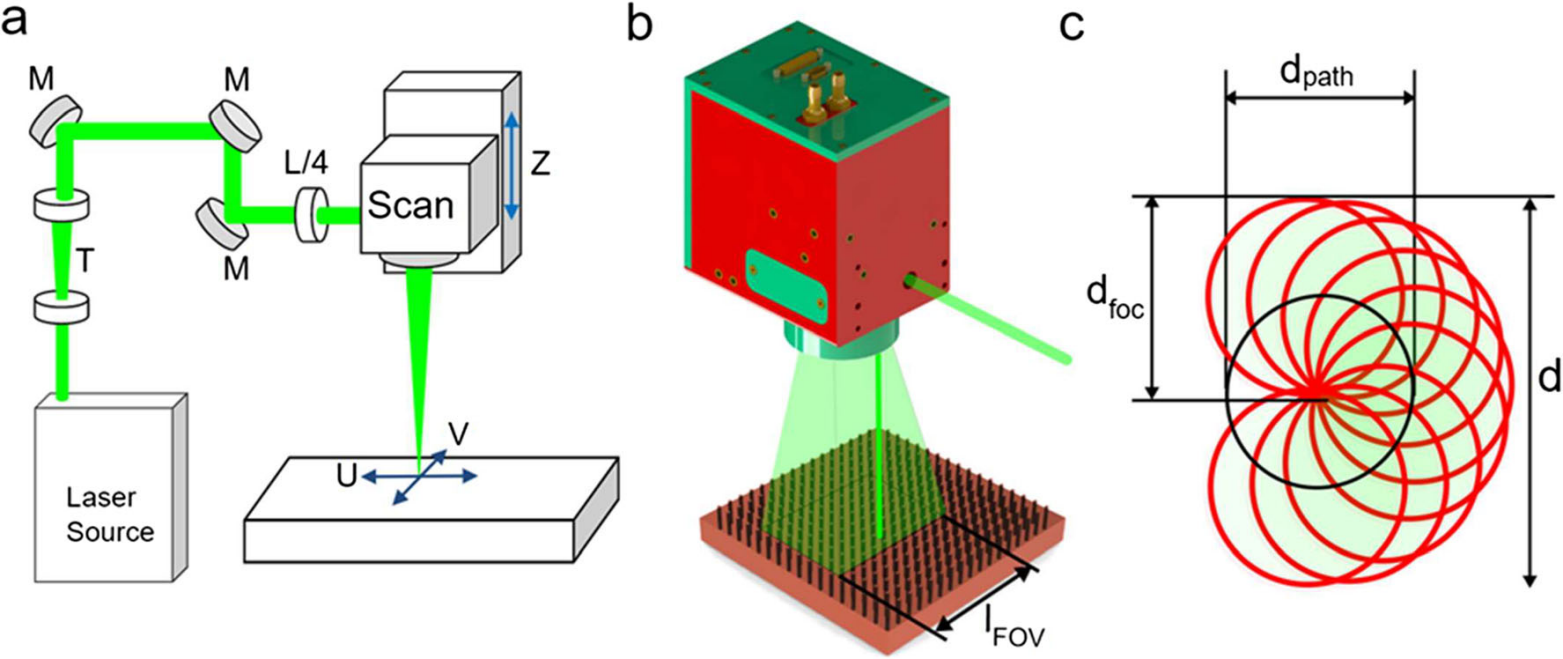
Schematic view of the laser setup (**a**). The laser beam is focused on the surface within the FOV with the side length l_FOV_ (**b**). Sketch representation of the circular path drilling strategy with corresponding control parameters (**c**)

The USP system used in this study is an Amphos 200 with 1 ps pulse duration, a wavelength of 1030 nm in the near-infrared light (NIR), and a beam quality of M^2^ < 1.3. With this USP system, a wavelength of 515 nm green light (GR) can be used by second harmonic generation. A repetition rate of 400 kHz at an output power of 2 W was selected for drilling the boreholes, and it was increased to 22 W for drilling the through-holes. Figure 1a shows schematically the setup with the laser source, a telescope (T), mirrors (M), and a waveplate (λ/4) generating a circular polarization state the two optical axes (U & V) and the focusing axis Z.

### Circular path laser drilling

The use of a scan head for laser ablation is a practical approach to control the laser beam highly dynamically and quickly. The focal length of the used f-theta objective lens is 163 mm, and the possible scanning speed is 1.5 m s^−1^. This allows the beam to move quickly in the focal plane within the field of view (FOV) of the scan head (see Fig. 1b). With 2.5D ablation, the material removal is generally performed in layers/planes. Such a layer consists of a frame that limits the ablation field and within this frame parallel paths that are followed by the laser beam during operation, forming a hatch. The next line is separated from the previous one by a hatching distance l_b_. The path does not necessarily have to be straight; it is also possible to follow any path, even the most demanding. In addition to the mirrors in the scan head, the path can also be aberrated by further mechanical axes and rotational axes. This makes it possible to remove even complicated structures such as conical helices from a cone by tangential machining [29]. It is important to note that the acceleration when changing direction also changes the speed. Due the speed reduction, the pulse overlap is increased which leads to an undesired discontinuous ablation.

For circular laser drilling, a circular path is used, where in contrast to percussion drilling, the exact beam shape has a smaller influence on the roundness of the drilled hole. Figure 1c shows the constant parameters of the strategy used to drill the holes, where the diameter of the circle corresponding to the path of the laser beam is termed *d*_*dpath*_ and the nominal focus diameter *d*_*foc*_ and *d* represent the total diameter of the surface irradiated with the laser of nominal focus diameter. For a given nominal focus diameter and desired diameter of the drill entry, the circle diameter of the path can be determined by

1 $$ {d}_{path}=d-{d}_{foc} $$

At the beginning of a drill, the beam is positioned in the middle of the diameter. Subsequently, the movement along the circular path of the laser spot is started and when the constant final speed is reached, the laser is turned on emitting laser pulses. In this way, a discontinuous removal is avoided and all the positioning in the FOV realized by optical axes movement in fast manner.

### Characterization method

X-ray tomography is a suitable examination method to explore the three-dimensional geometry of micro-holes. In this study, the micro-drillings were measured with an X-ray micro-computed tomography (μCT). The RX EasyTomXl Ultra 160 device by RX Solutions was used, which has an open nanofocus X-ray source operating at a voltage of 60 kV and a working current of 152 μA. The distance between the X-ray source and the detector is 400.204 mm, and the sample is positioned 3.141 mm behind the source. During a measurement, the sample is rotated a total of 1440 times; thus, it is possible to obtain a voxel size of 996 nm.

## Results and discussion

### Ablation characteristics

The ablation of OFC was studied with NIR and GR wavelength revealing a benefit for the green light in terms of efficiency. Copper shows a much lower reflectance at 515 nm compared with 1030 nm [30].

Moreover, shorter wavelengths enable a smaller focal spot diameter at enhanced Rayleigh length easing the processing without the need of refocusing. By examining the crater shape and its size after single laser pulse ablation, the threshold fluence can be determined using the equation established by Liu [31]. The rewriting of the formula to

2 $$ {D}^2=4{\mathrm{r}}_{\mathrm{equ}}^2=\alpha \ln {E}_p+\beta $$

allows a curve fit using the least squares method, where D is the diameter and *r*_equ_ the measured equivalent radius of the crater after single pulse ablation, *E*_*p*_ stands for the peak energy, and 𝛼 and 𝛽 are fitting parameters. This allows determining the diameter of the ablation crater represented as a function of the pulse energy and the threshold fluence derived by extrapolation over the axis intercept with the abscissa. In addition, the focal spot radius *w*_0_ can be estimated [31] with good agreement to measurements from the slope in a semi-logarithmic representation:

3 $$ {w}_0=\frac{\sqrt{\alpha }}{2} $$

**Fig. 2:**
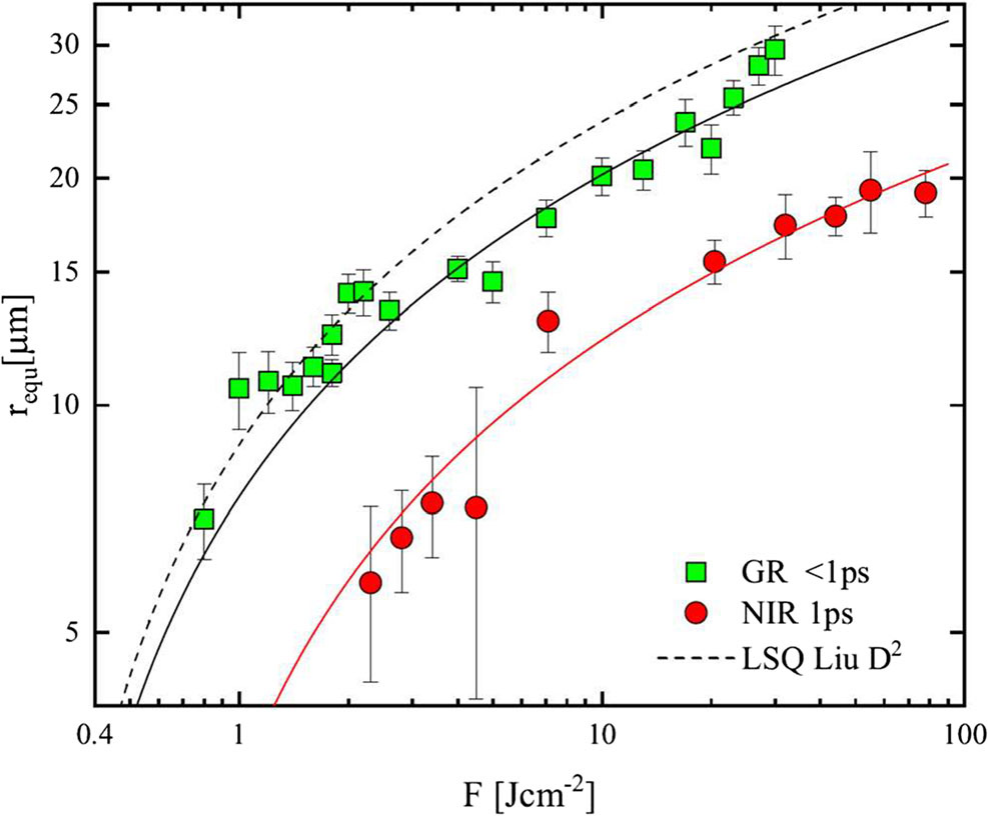
Measured equivalent radius plotted against the fluence for NIR and GR wavelength at 1 ps and LSQ fitting following the Liu plot

Figure 2 shows the measured equivalent radius plotted against the fluence for the test run with both wavelengths at 1 ps pulse duration. Additionally, the curve fitting according to Eq. (2) is shown. From the fluence dependence of the crater radius at GR wavelength, a value of 0.17 J cm^−2^ is obtained for the threshold fluence *F*_*th*_ and from the crater radius at NIR wavelength a value of 0.47 J cm^−2^. This is in good agreement with reported values from literature for picosecond pulses [32].

### Micro-hole drilling

In this study, the circular path drilling process was used to laser machine holes with an entrance diameter of approximately 35 μm and varying depths up to 165 μm (ratio 1:5) in a rapid manner leading to a small HAZ. The process was developed considering heat accumulation, and a recently proposed model allows to estimate the drilling depth that can be achieved for a given hole diameter at a material-dependent threshold and peak fluence [33]. This model is based on the assumption that the ablation during drilling stops naturally as soon as the local fluence falls below the threshold fluence due to projection at the evolving flanks. If considering a Gaussian beam, the diameter of the hole *d* for percussion drilling can be estimated as follows:

4 $$ d={w}_0\cdotp \sqrt{2\mathit{\ln}\frac{F_0}{F_{th}}} $$

where *F*_0_ is the applied peak fluence. If a percussion drilling method is used, where the focus remains at the same level, the result is a borehole with a positive cone angle. The energy of subsequent pulses is distributed over the developing conical geometry, and only the area where the incident fluence is above the threshold material is ablated. This correlation can be described as follows:

5 $$ {F}_{th}=\frac{E_p}{\frac{d}{2}\pi \sqrt{\frac{d^2}{4}+{h}^2}} $$

where ℎ describes the depth and following the denominator describes the shell surface of a cone. From the model in [33],

6 $$ h/{w}_0=\sqrt{\frac{F_0^2-{F}_{th}^2\mathrm{l}{\mathrm{n}}^2\frac{F_0}{F_{th}}}{2{F}_{th}^2\ln {F}_0/{F}_{th}}} $$

is established taking the large number of pulses into account at which the incubation is fully developed and the ablation process ends after infinite time. The maximum drilling depth ratio at a certain fluence ${F}_0=2{E}_p/{\pi w}_0^2$ can be estimated from (6), which for OFC with an exemplary fluence of 2.5 J cm^−2^, resulting in a ratio of 5 between radius and depth of the borehole. The holes should be drilled as quickly as possible and with no or small HAZ, whereby a low pulse repetition rate reduces heat accumulation and fast beam control reduces overlap in the proposed circular drilling process [34]. Due to these limitations, the process was developed at a fluence of 2.5 J cm^−2^ with a beam diameter in the focal plane of 10 μm, where the repetition rate was set to 400 kHz with an average power of 2 W. The laser followed the discussed circular path during drilling at 500 mm s^−1^. The circular path radius was set to 30 μm according to the hole diameter of 35 μm by compensating the beam radius by using Eq. (1).

### Geometrical evolution of blind holes

**Fig. 3:**
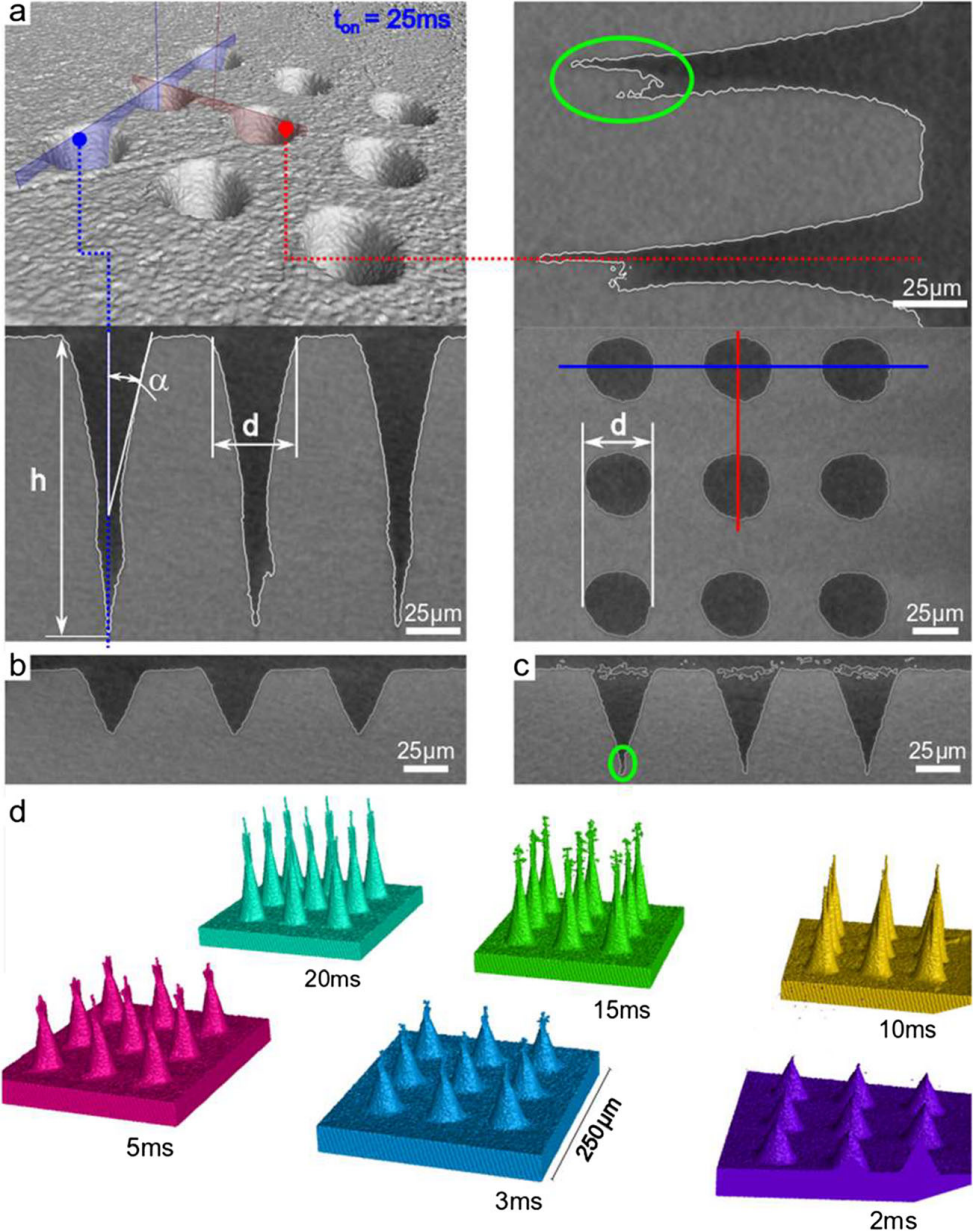
X-ray μCT measurements of circular path drilled boreholes with analysis markers giving key values. The borehole depth h, diameter d, and taper angle α at the entrance are determined (**a**). A set of nine holes per parameter are analyzed and show high geometric conformity within one set, where the transition from conical shape (**b**) to cylindrical and irregular can be observed (**c**). Exporting the inverse removed volumina (**d**) allows a distinct assessment of the geometry at certain times

The examination of the holes, drilled with the previously mentioned parameters, shows a good agreement between the developed hole geometry after drilling and the model behind Eq. (6). The drilling process was controlled within milliseconds at microsecond triggering time through the control emitting a certain number of pulses. The laser drilling time was varied between 2 ms and 35 ms, corresponding to 800 to 14,000 pulses impinging on the substrate. μCT was chosen to evaluate the drill hole geometry in a non-destructive and precise way. For one measurement, the 3D geometry of nine laser manufactured micro-holes per drilling time was analyzed. Figure 3a gives an overview of the analysis routine for drilled holes after 25 ms drilling time. The volume graph shown represents the 3D volume of the μCT measurement. By arbitrary segmentation and setting of measurement planes, the cross-section can be analyzed at any freely chosen point. From corresponding measurements, the drilling depth, the diameter, and the opening angle 𝛼 for all drilled holes were determined as shown in Fig. 3a.

A drilling time of 2 ms results in conical holes and axial cross section shown in Fig. 3b, with positive taper angle. The aspect ratio of drilling depth to input radius is slightly higher but in good agreement with the estimation from Eq. (6). This shows that the model for percussion drilling can also be applied for circular path drilling and gives good estimates. If the drilling time is increased by 1 ms and thus 400 additional pulses are added to one bore, additional material is removed as well from the flanks. As a result, the cone angle becomes more acute, the drilling depth increases, and irregular extensions start forming at the cone tip. These tails are marked green in Fig. 3a and c, and the formation occurs after reaching the conically shaped drill hole (Fig. 3b) geometry due to reflection of the laser radiation on the flanks. If the drilling time is increased further, the geometry in the initial area of the borehole seems to remain stable and conical. Following this section, a cylindrical geometry is formed, which becomes longer as the drilling time is further increased. Also, the irregular extensions at the end of the borehole remain in place and continuously reform. In Fig. 3d, the inverted volumes of the laser drilled holes are presented, which show the development of the hole geometry in more detail. It is expected that even deeper holes can be produced for very long drilling times, and, as reported in the literature, the typical smaller exit hole for through-hole laser drilling indicates a change in the hole geometry [13], resulting in cylindrical narrowing and formation of cavities. An off-axis bending of the laser beam at the evolving flanks changes the reflected part of the laser energy. Due to these multiple reflection in areas where the incident fluence is above the threshold fluence, an undesired ablation appears.

**Fig. 4:**
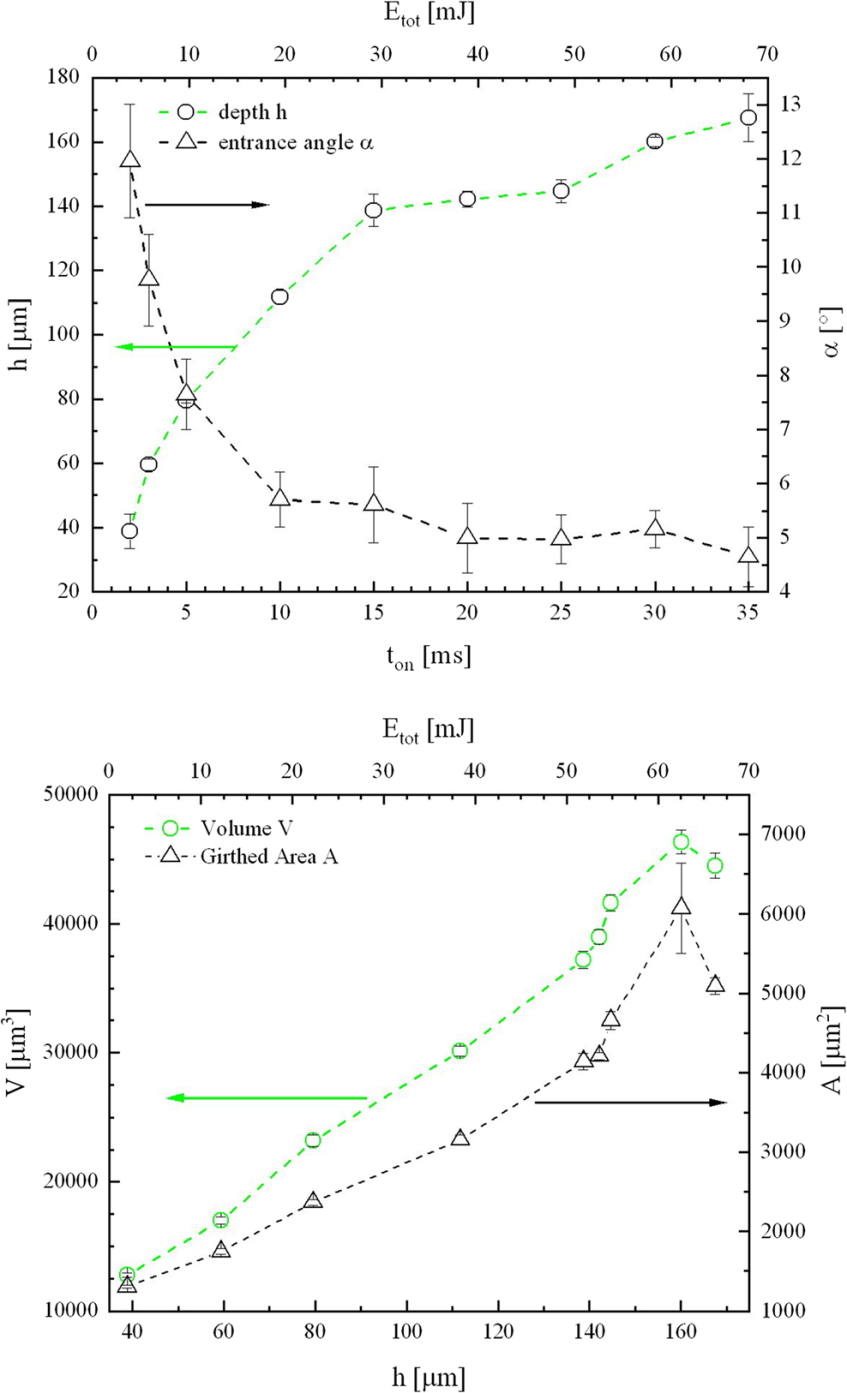
Key values for the development of micro drilling with increasing number of pulses and total energy per borehole. While drilling at 2 W and 400 kHz, resulting in 2.5 J cm^−2^, the depth increases with the number of pulses, and the taper angle decreases. After about 15 ms, saturation appears

The evaluation of the analyses for the drillings is summarized in Fig. 4, shown are the key values and their dependence on the drilling time and the total energy used per drilled hole. The depth of the boreholes saturates with increasing total energy/drilling time. This behavior is also evident for the cone angle that converges to 5° after 15 ms drilling time. The mantle surface of the drill hole (A, Fig. 4) increases more in comparison to the removed volume (V, Fig. 4). This illustrates the appearance of the cylindrical constriction and the irregular extensions at the tip of the hole.

The μCT proves to be a very practicable measurement technique to analyze geometries and possible cavities of laser-drilled holes, with a resolution in the micrometer range. The main advantage lies in the acquisition of the complete geometry, and in contrast to cross-sectional analysis with micrographs, there are no problems with cutting and grinding during sample preparation.

### Through-hole of 300 μm depth

**Fig. 5:**
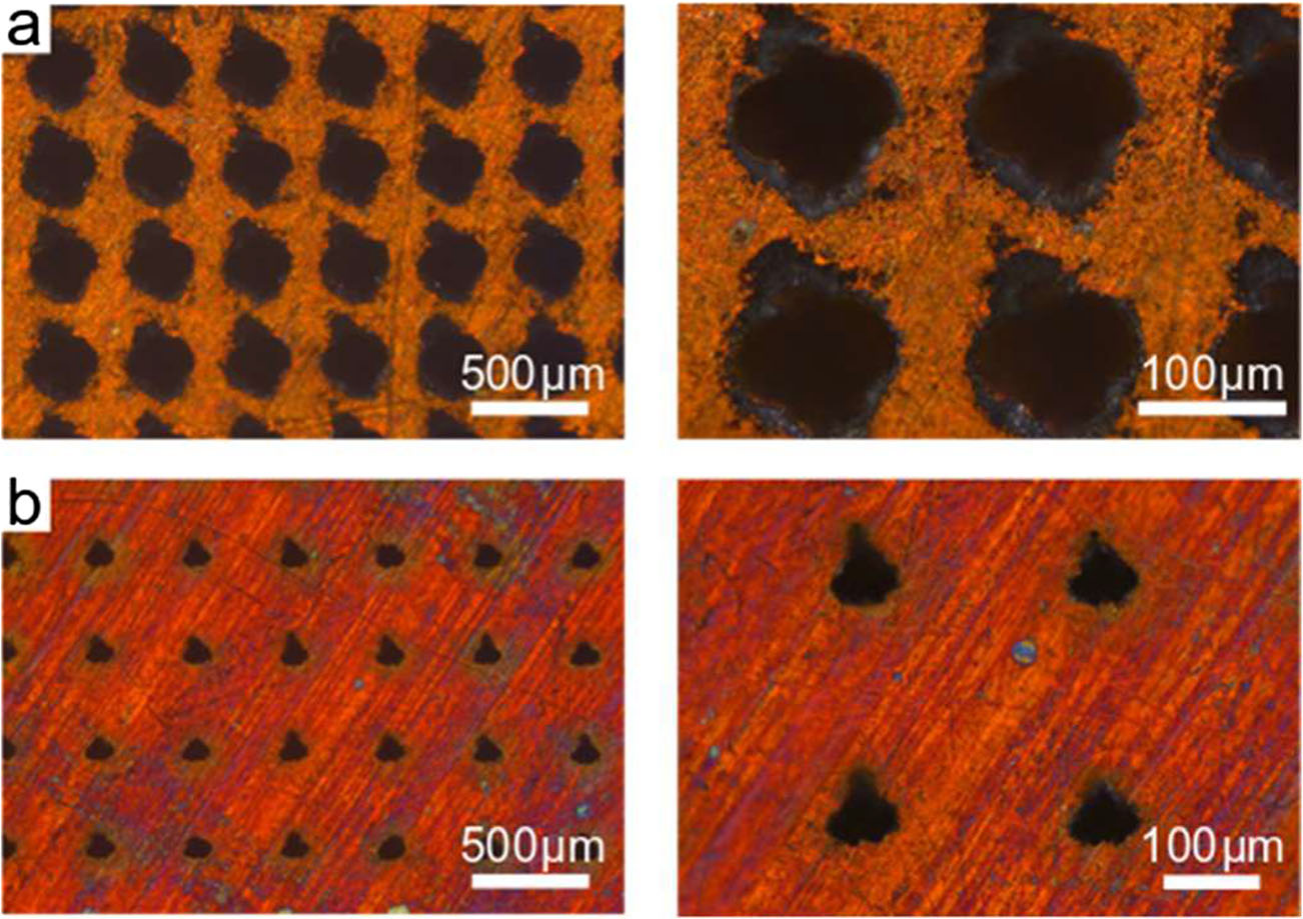
Microscopic images of the surface. Shown are the openings of the through-holes on the upper side at the inlet (**a**) and on the lower side at the outlet (**b**)

As already shown, the drilling depth saturates when the drilling time is increased. As a result, the process of drilling deeper in feasible time is no longer possible. One way to drill deeper holes is to use higher pulse energies and thus higher fluence or increase the drilling time. As an example, through-holes were processed in 300 μm thick OFC using the circular path method with an output power of 22 W and bigger spot diameter. Figure 5 shows the optical microscopic images of the through-holes. The input geometries, shown in Fig. 5a, have a diameter of roughly 50 μm. The drilling time per through-hole is 200 ms, which corresponds to 80,000 pulses. Taking into account the characteristic hole geometry, the assumption is plausible that here too a conical area is followed by a narrowed cylindrical part, as shown in Fig. 3. The diameter of the exit geometry is roughly 25 μm, observable in Fig. 5b. The shape of the input and output geometry is caused by the defectiveness of the laser pulses emitted from the source. However, the holes shown in Fig. 5 show high repeatability and a consistent entrance and exit geometry corroborated by the laser circular drilling of more than 92,000 holes.

## Conclusions and outlook

The results of the ablation study show advantages using green laser light over near-infrared laser light concerning laser drilling of copper. At the green wavelength, the threshold fluence is 2/3 lower, which means higher ablation at the same fluence and results in higher efficiency. In addition, the Rayleigh length increases extending the focus range and simplify the drilling process for higher aspect ratio.

Introducing μCT as a precise analytical method to study the development of the borehole shape at different drilling times proved to be very useful. A significant advantage is the non-destructive and detailed analysis of the total volume. The analysis of the boreholes has revealed the evolution of the borehole shape from conical at the beginning to steeper flanks forming a cylindrical shape after longer machining. This cylindrical constriction is formed adjacent to the cone and changes into irregular tails at the bottom of the borehole. The drilling depth saturates as the drilling time progresses, and a higher drilling depth can only be achieved by increasing the pulse energy. The measurement results from the μCT analysis and the estimates of the ratio of bore radius to bore depth from the used model show good agreement. This demonstrates the transferability of the model used for percussion drilling to circular drilling. It would certainly be interesting to carry out further investigations to explore the possible achievable depth by helical drilling and extend the model for a broader applicability.

### CRediT authorship contribution statement

Putzer Matthias Writing – original draft, Writing – review & editing.

Norbert Ackerl Conceptualization, Data curation, Formal Analysis, Funding acquisition, Investigation, Methodology, Project administration, Software, Visualization, Writing – original draft, Writing – review & editing.

Konrad Wegener Funding acquisition, Resources, Writing – review & editing.
